# The urological complications of vaginal birth after cesarean (VBAC) – a literature review

**DOI:** 10.25122/jml-2021-0219

**Published:** 2021

**Authors:** Valentin Nicolae Varlas, Yassin Rhazi, Roxana Georgiana Bors, Ovidiu Penes, Daniel Radavoi

**Affiliations:** 1. Department of Obstetrics and Gynaecology, Filantropia Clinical Hospital, Bucharest, Romania; 2. Department of Obstetrics and Gynaecology, Carol Davila University of Medicine and Pharmacy, Bucharest, Romania; 3. Department of Anesthesiology and Intensive Care, Bucharest Emergency University Hospital, Bucharest, Romania; 4. Department of Urology, Prof. Dr. Theodor Burghele Clinical Hospital, Bucharest, Romania

**Keywords:** VBAC, urological complications, uterine rupture, bladder rupture, ureteral lesion

## Abstract

The appearance of urological complications is a major problem in obstetrics and gynecologic surgery; the bladder is the most common damaged organ. Due to a continuous increase in the rate of cesareans, the incidence of urologic complications will be potentially higher. We reviewed the most important risk factors for urinary tract injury and analyzed the strategies necessary to avoid these situations during vaginal birth after cesarean (VBAC). The risks and benefits of VBAC should be balanced before deciding the mode of delivery.

## Introduction

Studies have shown that vaginal birth after low transverse cesarean section is considered safe and effective [1]. According to the Centers for Disease Control and Prevention (CDC), VBAC rates from the entire number of births increased from 12.4% in 2016 to 13.3% in 2018 [2]. After a previous cesarean section, the percentage of vaginal births in women who desired a VBAC is about 60–80% [3]. Due to the increased risk of uterine rupture, the induction of labor on the scarred uterus is controversial in the literature. Maternal and fetal complications can occur much more frequently during labor in the case of VBAC [4–6].

Every year, we are observing an increasing trend of cesarean deliveries, and the attention is focused on long-term complications. The increase in primary cesarean deliveries leads to a higher number of repeated cesarean deliveries [7]. In recent years, studies have shown a continuous increase in births by cesarean section; Romania is currently in first place in Europe with a rate of 46.9% [8]. The regulatory organizations are trying to compensate by promoting vaginal birth after cesarean section (VBAC), which is in its infancy in less developed countries. Achieving this goal is hampered by several restrictions on the reluctance of both physicians and patients regarding VBAC [9]. Despite this, VBAC was considered by many patients a good method to avoid repeated cesarean deliveries [10]. Attanasio *et al.* reported that at 12 months postpartum, 45% of women who gave birth by cesarean section want to experience VBAC for a future birth. Statistics prove the opposite by the fact that only about 12% would try VBAC [11].

Trial of labor after cesarean (TOLAC) is a planned or tried VBAC, being a safe option for many women in trying to reduce cesarean birth after cesarean (CBAC) [9].

In this review, we aimed to present the proper strategies for a successful VBAC and how to avoid potential urological complications.

## Tolac – Between Risk and Benefits

TOLAC is associated with a success rate ranging from 65% to 85% [12–14]. The rate of success depends on the previous indication for cesarean section (it is higher when the previous cesarean is done for malpresentation, gestational hypertension, or when the patient has a previous vaginal birth and may be lower when the cesarean is done for dystocia, diabetes, failure to progress or cephalopelvic disproportion) [15].

According to international guidelines (the American College of Obstetricians and Gynecologists – ACOG, Royal College of Obstetricians and Gynaecologists – RCOG, Le Collège National des Gynécologues et Obstétriciens Français – CNGOF), TOLAC should be taken into account in those medical units that have facilities regarding the management of unpredictable situations that may occur following a uterine rupture, and that may endanger the mother and fetus [16–18].

The main risk of TOLAC is uterine rupture which occurs in 0.2–0.9% of women with one previous C-section [10, 17, 19]. Uterine rupture was associated with maternal mortality in less than 1% of cases and perinatal mortality in 3 to 6% of cases [17]. The main risk factors of uterine rupture during TOLAC are an unknown previous uterine incision, an interpregnancy interval <12 months, poor wound healing of a previous uterine scar, and a prior preterm cesarean delivery [20].

Uterine rupture is an obstetrical emergency, causing an increased risk of fetal demise and severe maternal outcomes such as bladder and ureteral injury, especially when the patients are not well selected for VBAC [10]. Antepartum ultrasound evaluation of the uterine scar is insufficiently predictive of uterine rupture. Some authors report that a distance of 3.1 to 5.1 mm from the bladder wall to the amnion has a 96% sensitivity and a 63% specificity for the occurrence of a uterine defect (dehiscence, rupture). There is insufficient evidence to recommend the routine ultrasound assessment of uterine scar integrity [21, 22]. The cut-off value for the thickness of the uterine scar on the lower uterine segment measured by transvaginal sonography is not established. A thickness of the lower uterine segment less than 2.3 mm is correlated with an increased risk of uterine and urinary bladder rupture during TOLAC [23, 24].

The benefits of successful TOLAC derive from avoidance of the complications associated with cesarean delivery (immediate risks such as wound complications, injury to pelvic organs, and long-term risks such as abnormal placentation, uterine rupture, cesarean scar pregnancy, incisional endometriosis or adhesions) [10]. In pregnant women with a prior cesarean section (CS) with premature rupture of membranes (PROM), the induction of labor was observed to be accompanied by a double risk of uterine rupture and risk three times higher compared to the spontaneous initiation of labor [25].

## VBAC and Urological Complications

Uterine rupture is associated with the possible risk of bladder and ureteral injury [1]. The incidence of bladder injury associated with uterine rupture has a significant variability between 8% and 15% [3, 26]. The highest percentage is found after a failed VBAC trial [27]. Cahill *et al.* did not found any statistical difference regarding bladder lesions between VBAC and elective cesarean section (0.44% vs. 0.42%) [28]. Few cases of simultaneous rupture of the uterus and bladder at birth have been described [29, 30]. The clinical picture is diverse and includes hematuria (the most frequent symptom), severe lower abdominal pain, vaginal bleeding, palpation of fetal parts, cessation of uterine contractions, a non-reassuring fetal status, and maternal shock [31]. Other less common signs are vernixuria, oligo-anuria, and the presence of meconium in the urine [25, 29].

Urinary incontinence (stress urinary incontinence, urgency urinary incontinence, and mixed types of incontinence) was observed after vaginal delivery. Pregnancy itself is a risk factor for incontinence. Vaginal delivery has a major impact on the pelvic floor, weakening bladder neck support and affecting innervation [26]. It is estimated that approximately 12% of women report significant symptoms from stress incontinence and 8% from urgency incontinence. The risk of stress urinary incontinence is almost 2 times higher in women who delivered vaginally, with an increase in the absolute risk of 8% compared to cesarean section. The effect is more important in younger women as they have a longer life expectancy. The absolute risk of urgency incontinence is higher in women who deliver vaginally, with an increase of 3% compared to cesarean section [32].

We observed a heterogeneous incidence of bladder injuries during gynecological procedures compared to obstetric procedures. Some authors revealed a higher incidence in gynecologic interventions, and others showed the contrary. Thus, studies revealed an incidence of 0.49% for bladder injury, 0.24% for ureteral lesions in gynecological surgery, and 0.07–0.18% for bladder and 0.01–0.027% for ureter injury in obstetric surgery [23, 33, 34].

Bladder injury during cesarean section is reported in approximately 0.08 to 0.94% of cases [3]. When the rupture location was at the level of the uterine scar, characterized by poor vascularization, the hemorrhage did not occur. When CS is performed in the second stage of labor, the risk of incidental cystotomy is higher compared to the first stage of labor. In the particular case of a failed TOLAC, bladder injury was seen more often than in the control group (64% versus 22%, respectively) [3]. A higher rate of bladder ruptures is quickly recognized intraoperative and subsequently repaired with a significant decrease in patient morbidity [3].

Potential consequences of bladder injury include prolonged operative time, urinary tract infection, prolonged use of a bladder catheter, and vesicouterine or vesicovaginal fistula formation [3]. Long-term consequences of VBAC include an increased risk for urinary incontinence compared with nulliparous women or patients who underwent cesarean sections [10]. A thorough selection of the patients who can deliver vaginally after cesarean section may reduce, but not entirely cancel, some of these risks [35].

Existing data suggest that the presence of a prior cesarean delivery is associated with a 4-fold increased risk of bladder injury compared with a primary cesarean delivery. The incidence of bladder injury is higher in the group of patients who are subjected to emergency cesarean (careful dissection is not always a priority when attempting a fast delivery of a distressed fetus) and in the presence of uterine rupture [10]. Simultaneous uterine rupture and bladder injury are found in approximately 14% of cases [3]. After VBAC, Lua *et al.* showed a laparoscopic repair of uterine rupture using a two-layer technique simultaneous with rupture of the posterior bladder using a two-layer closure [36].

There are several other apparent risk factors for bladder injury at the moment of the cesarean section, such as age (bladder injury appears to be more frequent in older women), parity (bladder injury seems to be more frequent with increased parity), body mass index, adhesions, station of the presenting fetal part, time from skin incision to uterine incision, and estimated blood loss [31, 37]. The presence of adhesions in the abdominal cavity, particularly those between the bladder and the lower uterine segment, is associated with an increased risk of bladder injury during surgery (most bladder injuries occur during bladder dissection) [10]. Approximately 60% of patients with bladder injury had adhesions caused by a prior cesarean delivery compared with 10% in the control group. The density of intra-abdominal adhesions after cesarean is increasing and is related to the number of cesareans. Thus, the adhesion rates vary from 12–46% in patients with two CSs to 26–75% in those with three CSs [38, 39]. Risk factors for the development of adhesions include infection, excessive manipulation of tissue, increased blood loss during surgery, and tissue ischemia.

Most iatrogenic ureteral injuries are incomplete transection, but perforation (partial or complete), ligation, or complete transection can also occur. One review reported that 38% of ureteral injuries are initially missed. Long-term complications of missed ureteral injuries include urinoma, ileus, periureteral abscess, sepsis, ureteral fistula, or ureteral stricture [20].

A possible explanation for bladder-ureteral lesions may be the rupture of dense adhesions between the bladder and uterus, by high peritonization during the previous cesarean section, by an incongruence of the edges in multiple scarred uterus or associated endometriosis lesions [33, 40, 41]. Sharma *et al.* described a rare complication of uterine rupture associated with bladder rupture and ureter avulsion after vacuum-assisted VBAC [42]. Surgical conduct in such situations can be conservative (repair) or radical (hysterectomy).

Long-term complications of urinary tract injuries include urogenital fistulas. Obstetric fistulas are rare, most cases being identified after instrumental vaginal birth or in the case of manual extraction of the placenta. Most vesicouterine and vesicovaginal fistulas occur after cesarean section and uterine rupture. Postoperative urogenital fistula occurs due to direct lesions during dissection, being recognized during surgery and successfully repaired in most cases. Other possible causes of urogenital fistulas include injury by gripping or crushing, cauterizing, or kinking. In these cases, the blood supply to the tissues affected by necrosis should be reduced. The fistula may become symptomatic from days to months after surgery [43, 44].

Bladder injury must be recognized and repaired. Extravasation of urine into the peritoneal cavity causes peritonitis, ileus, and if prolonged, can lead to abdominal sepsis. Bladder injuries (intraperitoneal, extraperitoneal) are repaired using a two-layer technique. The first layer is closed with a running 3-0 absorbable suture. The second layer is closed using a running 2-0 or 3-0 absorbable suture. Urethral catheter drainage is recommended for two to three weeks. Prolonged bladder catheterization exposes the patient to an increased risk of urinary tract infection. The remains of the bladder, ureters, and bladder neck should be examined to exclude concomitant injury. Administration of agents that color the urine can assess the integrity of the lower urinary tract. A cystogram is done before catheter removal to evaluate healing. Other surgeons use one-layer sutures for cystotomies <2 cm and two layers for larger cystotomies. The bladder is epithelialized in three to four days and regains its normal function after 21 days. Additional antibiotic prophylaxis is not required when a urinary tract injury occurs, whether it is recognized intra- or postoperatively. Supratrigonal injuries are usually repaired with excellent results if they are recognized intraoperatively. On the other hand, injuries to the trigone or below the trigone may involve the ureters or urethra and are more challenging to repair than injuries at the level of the bladder dome or above the trigone. Exposure and suturing of this area are complex, and ureteral stent placement may be associated [3, 40].

During surgery, the most commonly used approach for ureteral lesions is to repair them by ureteroureterostomy or ureteroneocystostomy. It is preferable to perform ureteroureterostomy without a live anastomosis. The usual recommendation is to insert a ureteral stent following any repair that was performed. Ureteral lesions occur most frequently in the lower portion near the ureterovesical junction, ureteral anastomosis being possible if the lesion is located more than 3 cm from the ureterovesical junction. If the ureteral lesion is at a distance that is less than or equal to 2 cm from the ureterovesical junction, primary reconstruction is difficult, and reimplantation of the ureter (ureteroneocystostomy) is recommended [23].

## Discussion

It is difficult to evaluate the incidence of bladder and ureteral injuries after VBAC because there are few studies with no clinical trials. However, the low incidence of bladder and ureteral injury correlates to obstetricians' expertise of a tertiary center that encouraged VBAC. Another factor is represented by the level of training of obstetricians, some of whom also perform gynecologic oncology procedures.

Instead, the wrong selection of cases for TOLAC, intraoperative speed, history of cesarean section, fibroids, and endometriotic foci may increase the risk of injury of the bladder and ureters. In bladder lesions, the topography of the lesion is significant both in terms of its intraoperative resolution and long-term recovery. The ureteral injuries that might occur during cesarean result from intraoperative difficulty to control heavy bleedings from an anterior placenta site, a focal adherent placental tissue, an extension of the incision into the broad ligament, or blind hemostatic sutures. In very few cases, it's possible to damage the ureter directly following an iatrogenic extension of hysterotomy.

A higher incidence of left ureter damage was observed due to its anterior exposure by dextrorotation of the pregnant uterus [34]. Other author revealed no significant differences between the ureters regarding injury risk [20]. However, a study by Eisenkop and Rajasekar does not support this concept [23].

Because forensic reports showed frequent urinary tract injuries in recent years, the involvement of a urologist is essential to resolve ureteral injuries in particular.

Infant morbidity and mortality may be affected by the mode of delivery. Studies show that the infant mortality rates are higher in the case of TOLAC compared to repeated cesarean section (perinatal mortality rate of 0.13 versus 0.05 percent; neonatal mortality rate of 0.11 versus 0.06 percent). On the other hand, transient tachypnea of the newborn is more common in the case of repeated cesarean section compared with TOLAC (4.2 versus 3.6 percent, respectively) [45].

Counseling women with a prior cesarean section regarding the possibility of a vaginal birth should weigh its success in relation to potential maternal-fetal risks.

## Conclusions

Urinary tract complications are uncommon during the trial of labor after cesarean but carry the risk of serious morbidity. The risks and benefits of vaginal birth after cesarean should be balanced before deciding the mode of delivery. Both cesarean section and vaginal delivery are associated with the risk of urinary injuries. Vaginal delivery is more frequently associated with long-term side effects on the urinary tract, while the cesarean section is more often associated with injuries that are easily recognized and repaired during surgery with no long-term impact. Although many attempts have been made to successfully identify those patients who may benefit from a repeated cesarean section, no clinical or ultrasound criteria can predict with certainty the evolution of a trial of labor after a cesarean section.

## Acknowledgments

### Conflict of interest

The authors declare that there is no conflict of interest.
